# The effect of EMG magnitude on the masseter vestibular evoked myogenic potential (mVEMP)

**DOI:** 10.1016/j.joto.2022.06.004

**Published:** 2022-06-24

**Authors:** Daniel J. Romero, Gary P. Jacobson, Richard A. Roberts

**Affiliations:** Department of Hearing and Speech Sciences, Division of Vestibular Sciences, Vanderbilt University Medical Center, 1215 21st Ave. South, 6308 Medical Center East, Nashville, TN, 37232-8718, USA

**Keywords:** Vestibular evoked myogenic potentials (VEMPs), Masseter VEMPs, Electromyography (EMG), Vestibulomasseteric reflex (VMR), EMG target Level

## Abstract

**Introduction:**

The masseter vestibular evoked myogenic potential (mVEMP) is a bilaterally generated, electromyographically (EMG)-mediated response innervated by the trigeminal nerve. The purpose of the present investigation was to 1) determine whether subjects could accurately achieve and maintain a range of EMG target levels, 2) to examine the effects of varied EMG levels on the latencies and amplitudes of the mVEMP, and 3) to investigate the degree of side-to-side asymmetry and any effects of EMG activation.

**Methods:**

Subjects were nine neurologically and otologically normal young adults. A high-intensity tone burst was presented monaurally while subjects were seated upright and asked to match a range of EMG target levels by clenching their teeth. Recordings were made from the ipsilateral and contralateral masseter muscles referenced to the ear being monaurally stimulated.

**Results:**

We found that the tonic EMG target had no effect on mVEMP latency. Additionally, although mVEMP amplitudes “scaled” to the EMG target, there was a tendency for the subjects’ EMG level to “undershoot” the EMG target levels greater than 50 μV. While some individuals did generate differences in EMG activation between sides, there were no significant differences on average EMG activation between sides. Further, while average corrected amplitude asymmetry was similar across EMG targets, some individuals demonstrated large, corrected amplitude asymmetry ratios.

**Conclusions:**

The results of this investigation suggest that, as with cVEMP recordings, the underlying EMG activation may vary between subjects and could impact mVEMP amplitudes, yet could be mitigated by amplitude correction techniques. Further it is important to be aware that even young normal subjects have difficulty maintaining large, tonic EMG activity during the mVEMP recording.

## 1 Introduction

Vestibular evoked myogenic potentials (VEMPs) have become a component of the routine quantitative vestibular test battery. This has occurred, in large part, because VEMPs provide the only convenient modality to assess the integrity of the utricle and saccule. Although VEMPs can be recorded in response to mechanical, vibratory, and galvanic stimulation, investigators have overwhelmingly embraced high intensity acoustic stimulation for recording these signal-averaged responses. VEMPs can be evoked from several different muscle groups (Mohammed Ali et al., 2019), although are most commonly recorded from the sternocleidomastoid (i.e., cervical VEMP; cVEMP) and inferior oblique muscles (ocular VEMP; oVEMP; Colebatch et al., 1994; Rosengren et al., 2005).

In recent years, investigators have reported success in recording VEMPs from activated masseter muscles which has been referred to as the masseter VEMP (i.e., mVEMP; Deriu et al., 2005; Deriu et al., 2007). At present, it is not clear what part the mVEMP will play in clinical electroneurodiagnostic testing. It has been suggested that the mVEMP takes its peripheral end organ origins (i.e., the afferent limb of the reflex) from vestibular and cochlear receptors. The efferent limb of the reflex is mediated by the trigeminal system (Hickenbottom et al., 1985; Deriu et al. 2000, 2005, 2010).

### 1.1 Response characteristics

As occurred in the early stages of recording the cVEMP and oVEMP, research efforts have focused on developing a better understanding of the basic considerations for recording the mVEMP. The mVEMP is an inhibitory muscle reflex that scales with the underlying level of tonic electromyographic (EMG) activity (Deriu et al., 2005). To create a tonic level of EMG activation, the subject is asked to sit upright and clench their jaw which activates the left and right masseter muscles. A transient, high intensity acoustic stimulus is then presented monaurally through an audiometric earphone which is believed to produce stimulus-evoked fluid displacement of saccular hair cell bundles which activates the vestibulomasseteric reflex (VMR; Curthoys et al., 2015; Deriu et al., 2005). Activation of the VMR produces a stimulus-synchronized attenuation of masseter EMG resulting in the mVEMP.

The normal mVEMP appears as a biphasic deflection in the waveform consisting of an initial positive peak (i.e., occurring ∼11–12 msec) and a negative peak (i.e., occurring ∼21 msec). While the mVEMP shares a few similarities with the cVEMP (e.g., another inhibitory response whose amplitude scales with tonic EMG), the mVEMP has several unique characteristics that are not observed in the cVEMP or oVEMP. For example, the mVEMP is a bilateral symmetrical response that can be measured from either masseter muscle regardless of which ear is being monaurally stimulated (Deriu et al., 2007, Vignesh et al., 2021). Additionally, there is evidence that the mVEMP waveform has contributions from both vestibular and cochlear sensory systems. In this regard, the mVEMP consists of an initial vestibular component (p11/n15) that partially overlaps with a cochlear component (p16/n21; de Natale et al., 2019; Deriu et al., 2007).

### 1.2 Purpose

It is well-known that maintaining high levels of activated EMG over an extended period of time can be challenging for lay subjects and may represent a source of variability in VEMPs (Akin et al., 2004; McCaslin et al., 2014). Yet, it is currently unknown whether subjects can achieve and sustain a range of target EMG levels during mVEMP recordings. It is also unknown whether the levels of EMG activation differ significantly between the ipsilateral and contralateral masseter muscles. Lastly, it is unknown whether EMG activation levels influence mVEMP latency. Given the known impact of EMG on VEMP responses, a greater understanding of this characteristic is important as work continues to determine the clinical utility of the mVEMP.

The objectives of the current investigation were to: 1) determine whether normal subjects were capable of matching a range of EMG targets when activating the masseter muscles, 2) determine whether increases in EMG targets yield systematic increases in the amplitudes and latencies of the ipsilateral and contralateral mVEMP, and 3) to investigate the degree of side-to-side asymmetry and any effects of EMG activation.

## 2 Material and methods

### 2.1 Subjects

The study protocol was approved by Vanderbilt University Medical Center's Institutional Review Board (IRB# 211373). All subjects were consented by the investigators prior to being enrolled in the study. Nine young, and healthy subjects (3 males; 6 females) with a mean age of 27 years (SD = 3.4 years) were enrolled in the investigation. Subjects were excluded if they had any history of hearing loss or tinnitus. Further, subjects were excluded from participation if they reported any history of dizziness or balance impairment or if they reported a history of middle ear or neurological disease. Lastly, one subject was excluded from participation for failing to generate an mVEMP during data collection.

### 2.2 Stimulus

A 500 Hz Blackman-gated tone burst with a 4 msec duration (2-0-2) was delivered with ER3A insert earphones at 125 dB peak-sound pressure level (dB pSPL) with a stimulus rate of 5.4 per second. The stimulus level used in this study was comparable to the stimulus levels used for the recording of the cVEMP and oVEMP during clinical testing (Rosengren et al., 2019). We ensured that the stimulus intensity level over the duration of data collection for each subject did not exceed 100% of the recommended daily dose of noise exposure recommended by the National Institute for Occupational Safety and Health (NIOSH; Portnuff et al., 2017).

Calibration of the stimulus was performed using a Larson Davis 824 sound level meter attached to a ½ inch microphone and 6 cc coupler. Adjustments were made to the dial of the attenuator to ensure the stimulus level was being delivered at the desired physical level (125 dB pSPL).

### 2.3 Procedure

Stimuli were presented monaurally. The ear receiving the stimulus was counterbalanced across subjects. Data were collected using Neuroscan SCAN software (Version 4.5). Subjects were seated in a comfortable recliner chair in an upright position. A two-channel electrode montage was employed consisting of four (4) Ag/AgCl disposable electrodes. The electrodes were applied to the surface of subject's skin using clean electrode preparation techniques. Two (2) non-inverting electrodes were applied to the belly (i.e., the lower third) of the left and right masseter muscles which were identified by having the subject clench their jaw. One (1) inverting electrode was placed over the zygomatic arch (i.e., ipsilateral to the ear being monaurally stimulated) approximately 3 cm superior to the non-inverting electrode (Loi et al., 2020). One (1) ground electrode was then placed on the forehead (i.e., Fpz). Individual electrode impedances were below 5 kΩ and no interelectrode impedances were greater than 3 kΩ.

EMG signals were amplified (X2000), filtered 5–1500 Hz, and sampled at a rate of 10 kHz, and signal averaged over a 100 msec epoch including a 20 msec prestimulus period (i.e., the data were signal averaged 20 msec prior to and 80 msec following stimulus onset). A minimum of 128 sweeps were collected for each recording. The EMG was monitored at five target levels of muscle contraction (i.e., no contraction [rest], 30, 50, 100, and 150 μV). EMG monitoring was accomplished using a dedicated evoked potential system (Intelligent Hearing Systems, Smart EP; Version 5.20). Subjects were provided with a real time bar graph displaying rectified EMG from the left and right masseter muscles. Two black target lines were placed on either side of the desired target level (+/- 10 μV) and subjects were instructed to maintain EMG activation as close as possible to the target level (+/- 10 μV) throughout the duration of each run. EMG levels that fell outside this range (+/- 10 μV) were still accepted in the data collection. Given the stimulus rate, each run required ∼25 s to record 128 sweeps. Conditions were randomized to minimize the effect of muscle fatigue and a rest period of at least 30 s was provided for each subject between runs.

### 2.4 Analysis

Evoked response peaks were identified by an experienced examiner (DR) using a custom MATLAB program. The latencies and raw/corrected peak-to-peak amplitudes were labeled. Corrected peak-to-peak amplitudes were calculated by dividing the peak-to-peak amplitude by the mean rectified EMG amplitude. A present mVEMP was operationally defined as a positive peak occurring ∼11–12 msec followed by a negative peak occurring ∼21 msec after stimulus onset (Deriu et al., 2007). Actual EMG activation levels were calculated offline using the mean rectified EMG during the prestimulus baseline (i.e., −20 to 0 msec). The same analysis was performed for both the ipsilateral and contralateral channels. Furthermore, corrected amplitude asymmetry between the ipsilateral and contralateral sides was calculated using the following equation: $\left(\frac{larger\;amp-smaller\;amp}{larger\;amp+smaller\;amp}\right)\times 100$

### 2.5 Statistical approach

Data were analyzed using SPSS, version 27.0 (IBM SPSS Statistics for Windows, Armonk, New York). An alpha level of 0.05 was chosen for all analyses. Descriptive statistics including mean p1, and n1 latencies, p1 amplitudes, raw and corrected peak-to-peak amplitudes, and EMG activation level are reported. A paired-samples*t*-test was performed to determine whether significant differences existed between the ipsilateral and contralateral recordings. Individual and average EMG from the ipsilateral and contralateral masseter muscles were compared to each target level. A two-way, repeated measures ANOVA was conducted with within-subject factors of EMG target level (4 EMG target levels) and masseter muscle (2 levels, ipsilateral and contralateral recordings) was used to determine the effect of EMG activation on p1 and n1 latencies, p1 amplitudes, and raw/corrected peak-to-peak amplitudes. Further, a linear regression analysis was conducted to further describe the relationship between EMG and raw peak-to-peak amplitude of the mVEMP.

## 3 Results

### 3.1 Waveform analysis

**Fig. 1:**
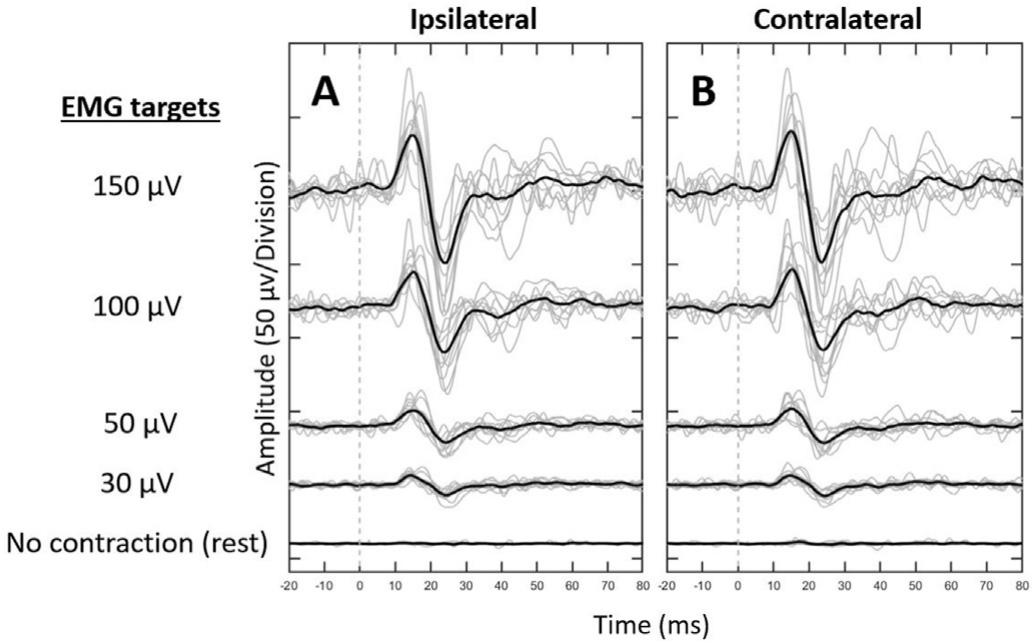
Individual (grey traces) and grand average (black traces) mVEMP waveforms for each EMG target level for the ipsilateral (**A**) and contralateral (**B**) masseter muscles across all subjects. Note that positive deflections at the active electrode is going up.

Fig. 1 shows the grand averaged and individual mVEMP waveforms for the ipsilateral and contralateral masseter muscles across all EMG target levels (i.e., rest, 30, 50, 100, and 150 μV). The relationship between EMG activation and peak-to-peak amplitude was consistent with previous mVEMP studies (Deriu et al., 2005). Larger but more variable peak-to-peak amplitudes were collected from higher levels of EMG activation. Smaller peak-to-peak amplitudes were observed at lower EMG target levels. There were no visually identified responses when subjects were at rest (no muscle contraction). Since no response was identified at rest, results from those conditions were not included in the statistical analysis. Further, all subjects generated a bilateral mVEMP with the exception of one subject during the 50 μV condition; this subject was excluded from the statistical analysis. The descriptive mVEMP data for each EMG target level is shown in Table 1 for p1 and n1 latency, raw and corrected p1-n1 amplitude, and EMG activation for the ipsilateral and contralateral masseter muscles across all subjects.

**Table 1:** Means and SDs of mVEMP p1 and n1 latencies, p1 amplitude, raw/corrected p1n1 amplitude, and mean rectified EMG across EMG target level.

	*n*	EMG Target (μV)	Average EMG (μV)	p1 Latency (ms)	n1 Latency (ms)	p1 amplitude (μV)	p1-n1 (μV)	Corrected p1-n1
**Ipsilateral**	9	150	117.4 (20.1)	14.7 (1.6)	23.1 (1.4)	43.8 (15.5)	106.2 (31.5)	1.0 (0.3)
	9	100	77.6 (8.5)	14.7 (1.5)	23.1 (1.4)	30.0 (8.3)	69.2 (20.1)	0.9 (0.3)
	9	50	38.9 (1.6)	14.8 (1.5)	23.4 (1.1)	13.7 (5.3)	29.3 (10.2)	0.7 (0.3)
	7	30	23.6 (1.9)	14.7 (1.9)	23.7 (1.9)	8.2 (2.2)	17.0 (4.8)	0.7 (0.2)
	0	No contraction	4.2 (2.3)					

**Contralateral**	9	150	117.3 (22.8)	14.7 (1.4)	23.9 (0.5)	43.4 (21.6)	105.4 (43.8)	0.9 (0.4)
	9	100	82.0 (20.4)	14.8 (1.2)	23.5 (0.8)	32.1 (13.1)	69.9 (29.1)	0.9 (0.4)
	8	50	39.7 (7.9)	15.1 (1.6)	23.5 (1.1)	14.2 (6.0)	32.1 (11.9)	0.8 (0.3)
	7	30	25.7 (4.9)	15.7 (1.7)	24.5 (0.8)	8.8 (3.2)	18.3 (7.0)	0.8 (0.3)
	0	No contraction	6.0 (2.5)					

### 3.2 EMG activation

**Fig. 2:**
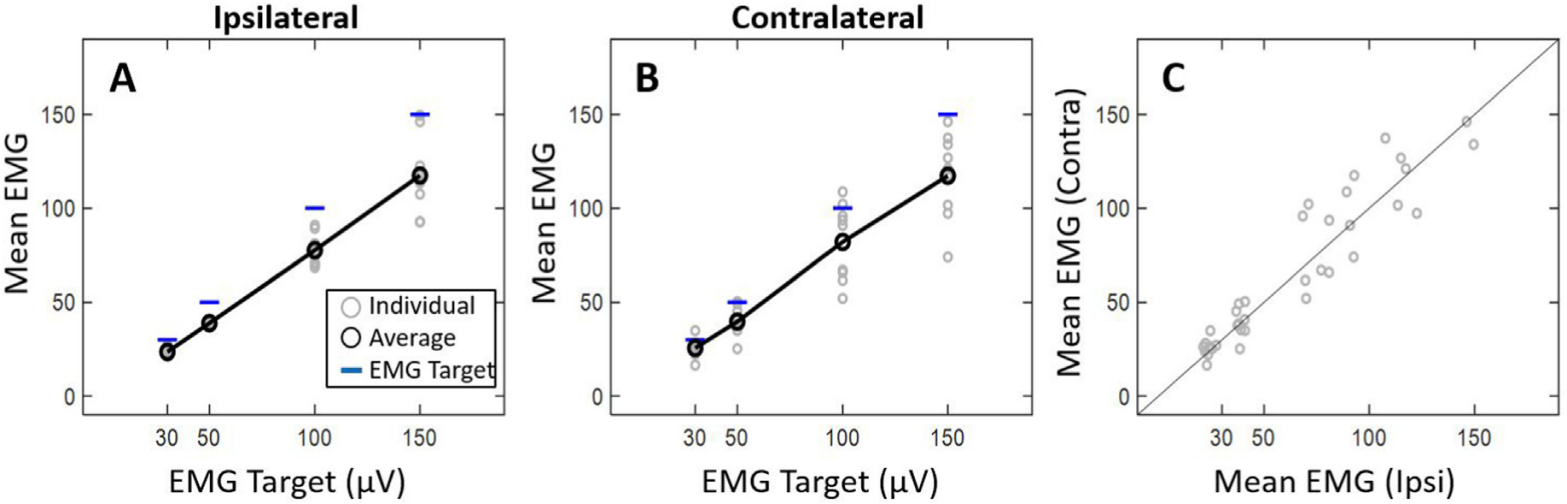
Bivariate plots demonstrating the relationship between EMG activation and desired EMG target level. Individual (grey) and mean (black) EMG activation relative to the EMG target level (blue) for the ipsilateral (**A**) and contralateral (**B**) muscles. Ipsilateral and contralateral muscles are also compared (**C**).

Fig. 2A–B displays the individual and average EMG activation that was for the ipsilateral and contralateral sides across EMG targets (30–150 μV). On average, subjects produced EMG activation less than the target level. This difference between actual EMG and the EMG target increased at higher levels of muscle contraction. Likewise, inter-subject variability in matching EMG targets increased with EMG target level. This was true for the ipsilateral and contralateral sides. Fig. 2C compares mean EMG from the ipsilateral muscle (i.e., ipsilateral to the stimulated ear) to the mean EMG from the contralateral muscle. A paired samples *t*-test revealed that the EMG activation between the ipsilateral and contralateral sides was not different (*p* > .05). That is, regardless of the muscle being measured, there was a tendency to “undershoot” the desired EMG target. The inter-subject variance increased with EMG target level for both muscles.

**Fig. 3:**
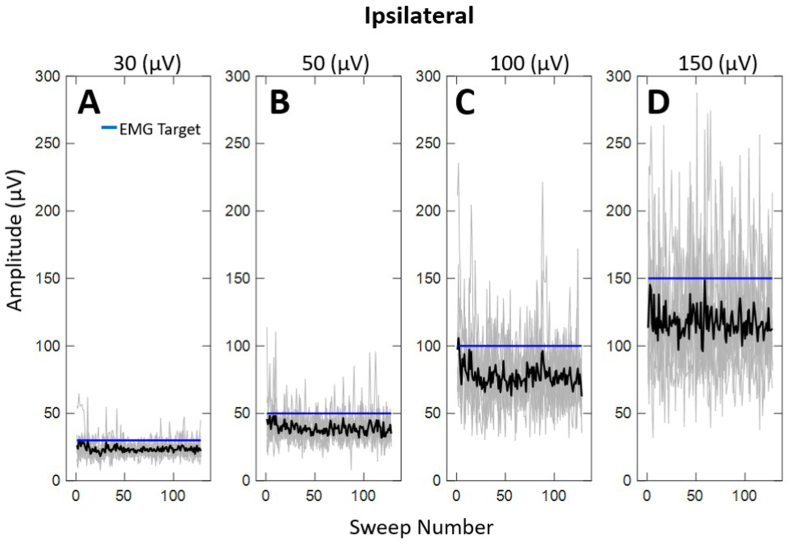
Individual (grey traces) and average (black traces) EMG activation across EMG target level for the ipsilateral side to the ear stimulated across all subjects.

**Fig. 4:**
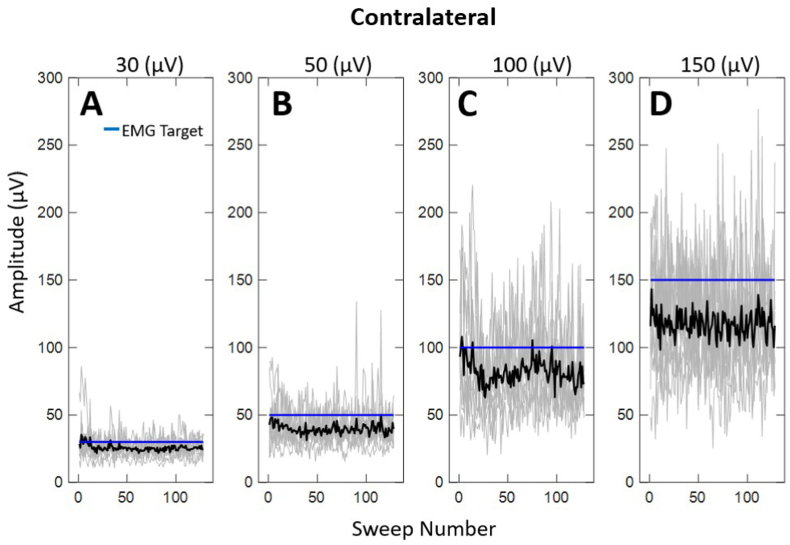
Individual (grey traces) and average (black traces) EMG activation across EMG target level for the contralateral side to the ear stimulated across all subjects.

Given the difficulty subjects experienced in matching EMG targets, we were interested in determining if subjects reached the EMG targets for any stimulus sweeps. Fig. 3, Fig. 4 illustrate the rectified EMG activation across each stimulus sweep (i.e.,128 sweeps for each signal average) for the ipsilateral and contralateral recordings, respectively. Similar to EMG levels averaged across sweeps, subjects achieved EMG activation less than the EMG target, especially for higher levels of muscle contraction. Likewise, subjects demonstrated greater variability attempting to match the higher EMG target levels. Similar to mean EMG, there was no significant difference in the rectified EMG activation across sweeps between the ipsilateral and contralateral sides (*p* > .05). Additionally, the rate at which participants reached the EMG target was dependent upon the level of muscle contraction. (Fig. 3, Fig. 4A, D).

**Fig. 5:**
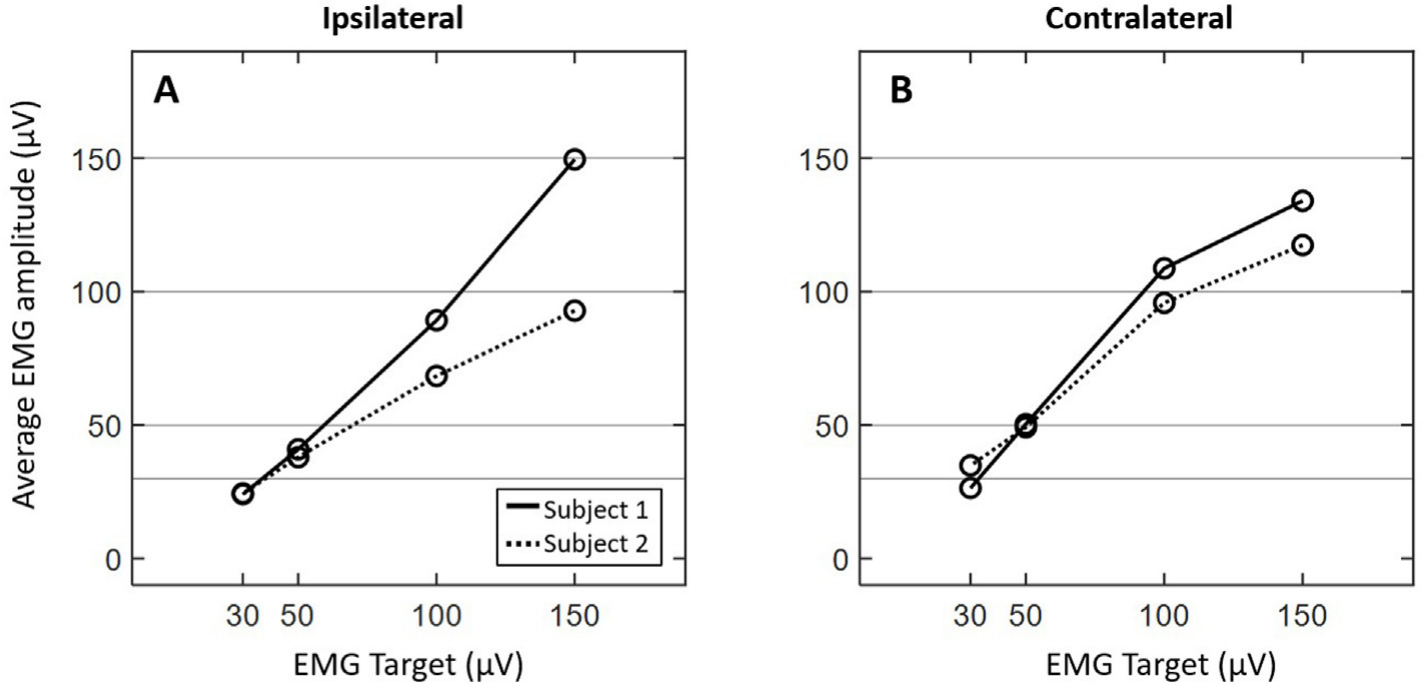
Example EMG activation across two individual subjects for the ipsilateral (**A**) and contralateral sides (**B**). Each target level is designated by horizontal lines on the ordinate.

While subjects on average experienced a greater difficulty reaching higher levels of EMG activation, this also varied between subjects. Fig. 5 displays the average EMG activation of two subjects across all EMG target levels. Subject 2 experienced a greater amount of difficulty reaching the target level when compared to Subject 1. Additionally, average EMG activation between the ipsilateral and contralateral masseter for each subject also showed variation. That is, the ability to reach the target level was dependent upon the masseter muscle in some individuals (Fig. 5A and B).

### 3.3 Latency

**Fig. 6:**
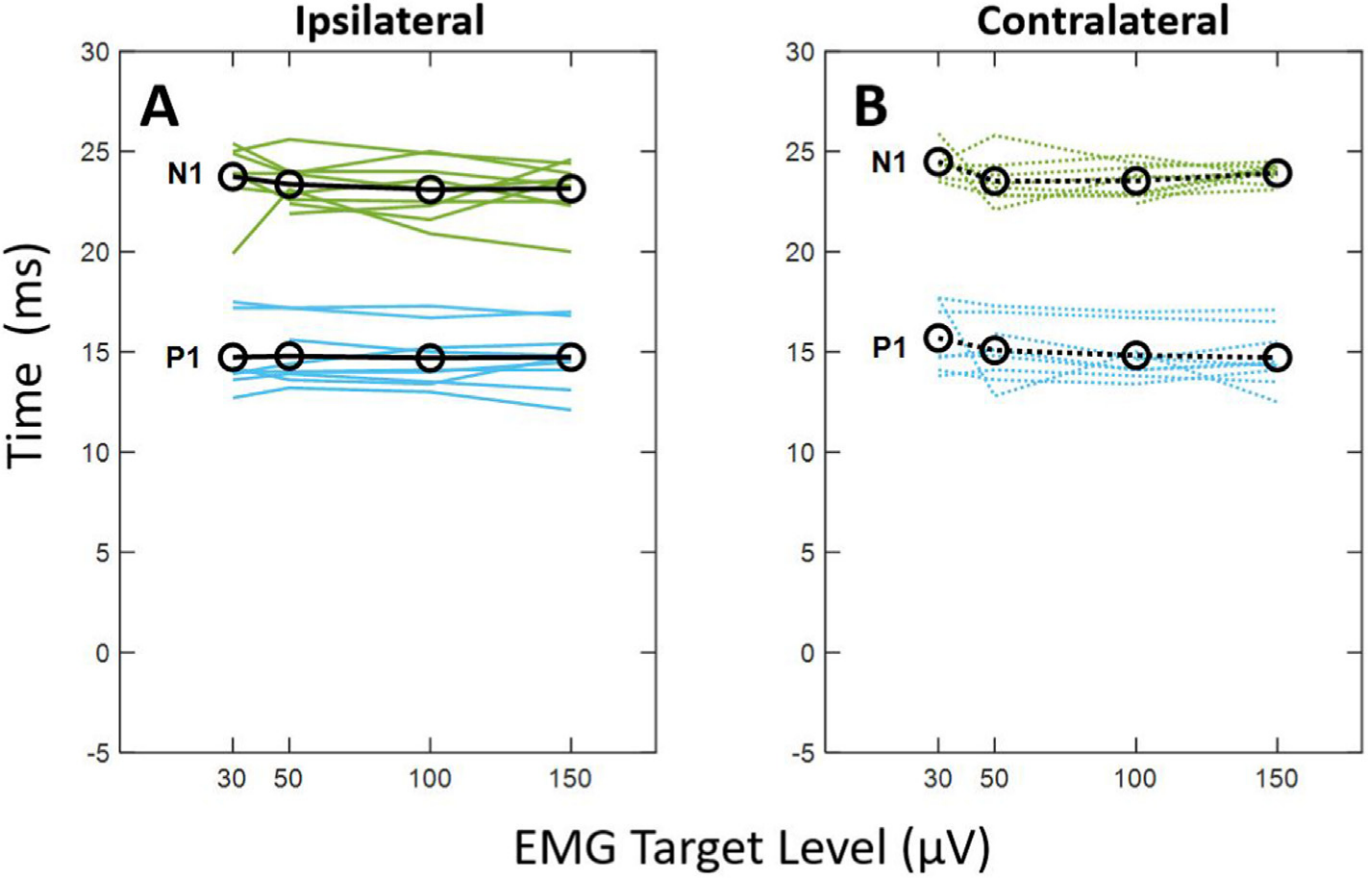
Individual and average P1 (blue) and N1 (orange) latencies across EMG target level for the ipsilateral (**A**) and contralateral (**B**) sides across all subjects.

The main effects for EMG target level on p1 latency (F(1.3, 8.0) = 1.039, p = .364) and n1 latency (F(1.8, 11.0) = 3.227, *p* = .082) were not statistically significant. There was also no significant main effect of masseter muscle side (ipsilaterally versus contralaterally) on p1 latency (F(1, 6) = 1.058, *p* = .343) or n1 latency (F(1, 6) = 1.309, *p* = .296). The interaction between EMG target level and masseter muscle side on p1 latency (F(1.4, 8.9) = 1.646, *p* = .242) or n1 latency (F(1.1, 6.9) = 1.154, *p* = .330) was not statistically significant. As expected, latencies were similar across EMG target levels for the ipsilateral and contralateral muscles (Fig. 6A and B).

### 3.4 Raw peak-to-peak amplitude

Given the prior observations vestibular and cochlear contributions to the mVEMP waveform, it was important to ensure that p1 amplitude (vestibular component) was positively correlated with peak-to-peak amplitude. A Pearson-product correlation showed that there was a strong positive correlation between p1 amplitude and peak-to-peak amplitude for the ipsilateral [r(33) = 0.861, *p* < .001] and contralateral sides [r(33) = 0.875, *p* < .001]. Furthermore, p1 amplitude strongly predicted peak-to-peak amplitude as shown through linear regression for the ipsilateral [R^2^ = 0.741, F(1, 32) = 91.437, *p* < .001, y = 2.065x + 6.385] and contralateral sides [R^2^ = 0.765, F(1, 32) = 101.111, *p* < .001, y = 2.004x + 7.518]. Given this strong relationship between p1 amplitude and peak-to-peak amplitude, all subsequent analyses were performed on peak-to-peak amplitude.

**Fig. 7:**
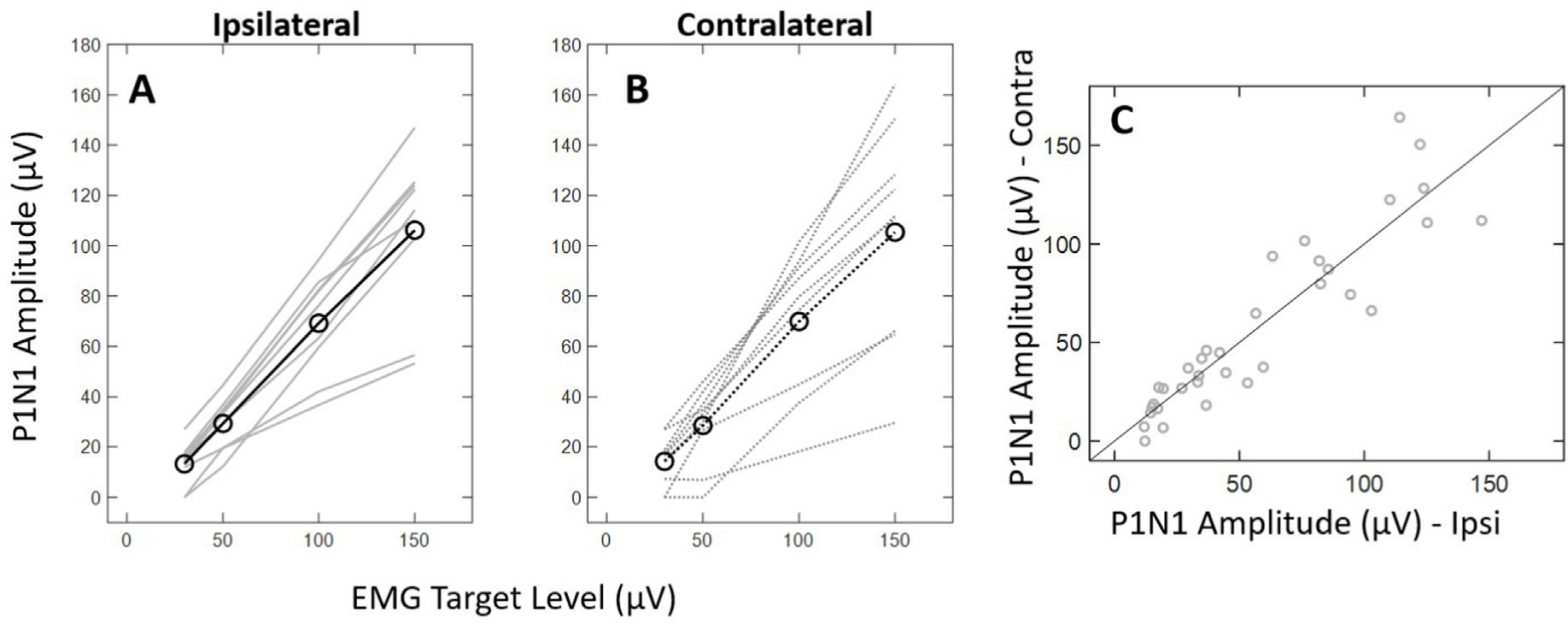
Individual and average raw peak-to-peak amplitudes across EMG target level for the ipsilateral (**A**) and contralateral (**B**) sides across all subjects. Ipsilateral and contralateral peak-to-peak amplitudes are also compared (**C**).

A 4x2 within-subjects repeated measures ANOVA revealed a significant main effect of EMG target (F(1, 6.5) = 67.375, *p* < .001, ηp2 = 0.918) on peak-to-peak amplitude. Post-hoc tests using Bonferroni corrections for multiple comparisons were performed and revealed that peak-to-peak amplitude at all EMG targets were significantly different from one another (*p* < .01). The main effect of masseter muscle side (F(1, 6) = 0.123, *p* = .738) was not statistically significant. There was not a significant interaction between EMG target level and masseter muscle side for peak-to-peak amplitude, (F(1.1, 6.8) = 0.123, *p* = .769). Fig. 7 shows the raw peak-to-peak amplitudes across EMG target levels and and are summarized in Table 1. On average, mVEMP raw peak-to-peak amplitudes significantly increased as EMG activation increased (*p* < .001) for the ipsilateral and contralateral masseter muscles (Fig. 7A and B). In addition, the differences between ipsilateral and contralateral amplitudes were not significant (*p* > .05; Fig. 7C).

### 3.5 EMG and raw/corrected peak-to-peak amplitude

**Fig. 8:**
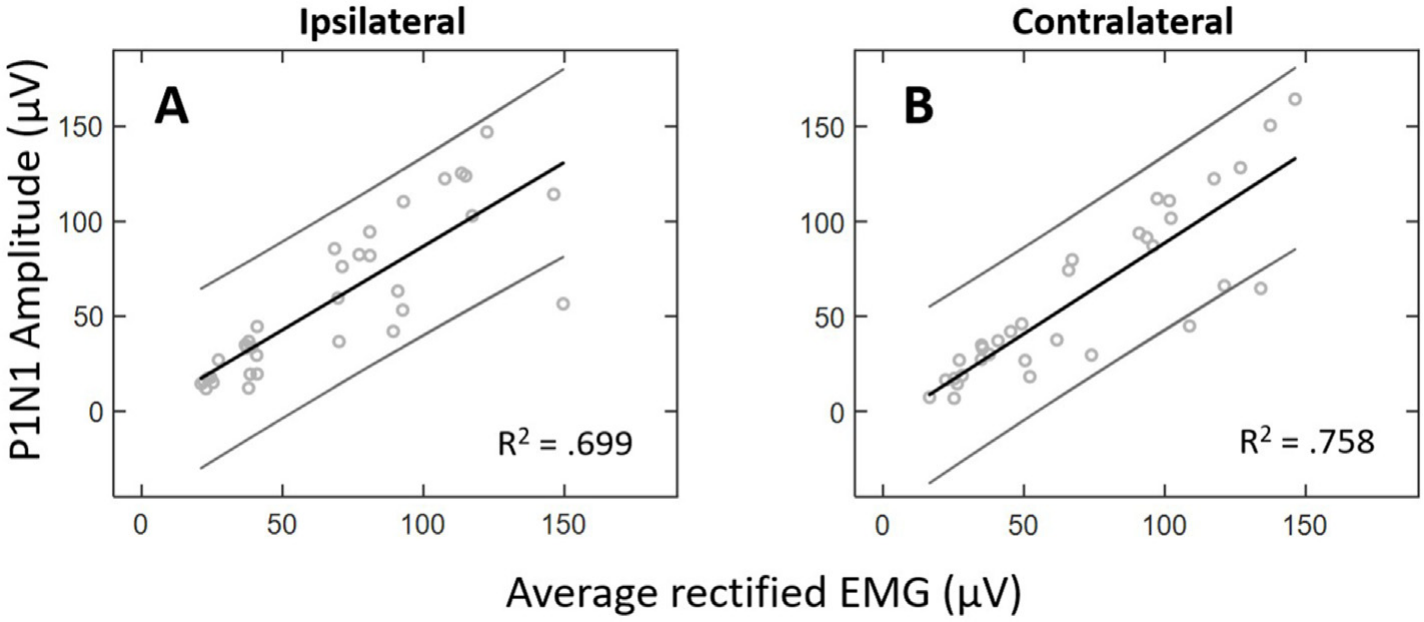
Bivariate plot demonstrating the relationship between average EMG and raw peak-to-peak amplitudes for the ipsilateral (**A**) and contralateral (**B**) sides across all subjects. The 95% confidence interval to give a range of gradients is also shown on either side of the regression slope.

To further describe the relationship between EMG and peak-to-peak amplitude, a simple linear regression was performed and revealed that EMG amplitude was a significant predictor of raw peak-to-peak amplitude for the ipsilateral [R^2^ = 0.699, F(1, 32) = 74.355, *p* < .001, y = 0.882x - 1.245] and contralateral sides [R^2^ = 0.758, F(1, 31) = 97.056, *p* < .001, y = 0.958x - 7.120]. That is, as EMG activation increased, there was a significant increase in peak-to-peak amplitude (shown in Fig. 8). A paired-samples*t*-test indicated no significant difference in the slopes between the ipsilateral and contralateral sides (*p* > .05). The relationship between EMG activation and peak-to-peak amplitude for the ipsilateral and contralateral sides is shown below (Fig. 8).

**Fig. 9:**
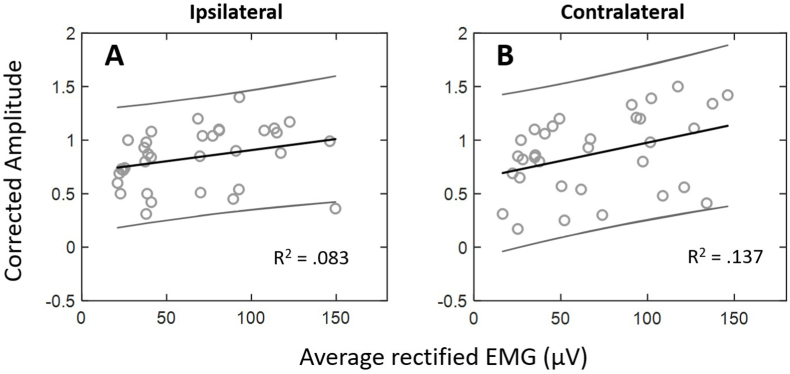
Individual and average corrected peak-to-peak amplitudes across EMG target level for the ipsilateral (**A**) and contralateral (**B**) sides across all subjects. The 95% confidence interval to give a range of gradients is also shown on either side of the regression slope.

Fig. 9 shows the corrected amplitude data across EMG target levels for the ipsilateral and contralateral muscles. On average, corrected mVEMP amplitudes were similar across EMG target levels and the two sides were positively correlated with one another [r(33) = 0.791, *p* < .001]. As expected, simple linear regression revealed that EMG amplitude was a weak predictor of corrected peak-to-peak amplitude for the ipsilateral [R^2^ = 0.083, F(1, 32) = 2.892, *p* = .099, y = 0.002x + 0.700] and contralateral sides [R^2^ = 0.137, F(1, 31) = 4.906, *p* < .034, y = 0.003x + 0.637]. A paired-samples*t*-test revealed a significant difference between in slopes between the ipsilateral and contralateral sides (*p* = .014). The range of corrected amplitudes for the ipsilateral side was 0.5–1 at 30 μV, 0.31–1.08 at 50 μV, 0.45–1.09 at 100 μV, and 0.36–1.17 at 150 μV. The range of corrected amplitudes for the contralateral side was 0.31–1.1 at 30 μV, 0.17–1.13 at 50 μV, 0.25–1.39 at 100 μV, and 0.3–1.42 at 150 μV.

Amplitude asymmetry ratio is typically used to compare the percentage difference between the left and right ears. This study did not compare ears (the stimulus was monaurally presented) but rather analyzed the amplitude asymmetry ratio between the ipsilateral and contralateral sides to determine the average percentage difference between sides. The average corrected amplitude asymmetry ratio was 8.9% [range = 0–23.5%] at 30 μV, 12.3% [range = 0–49%] at 50 μV, 12.9% [range = 0–34%] at 100 μV, and 12.3% [range = 1.8–28%] at 150 μV. A one-way ANOVA did not reveal a significant effect of EMG target level on corrected amplitude asymmetry ratio (F(2.2, 20.5) = 0.508, *p* = .633).

## 4 Discussion

The purposes of this study were: 1) to determine whether it was possible for normal subjects to match a range of masseter EMG targets, and 2) determine the effects of varied EMG target levels on the latency and amplitude of the ipsilateral and contralateral mVEMP, and 3) to investigate the degree of side-to-side asymmetry and any effects of EMG activation.

Most interestingly, subjects tended to “undershoot” the EMG target throughout the mVEMP recording, especially at higher levels of EMG activation. The gap between average EMG levels and the target level was greater when the target exceeded 50 μV (Fig. 3, Fig. 4). Another significant finding was that EMG was more variable at higher levels of muscle contraction. In this study, mVEMP latencies were unaffected by varying levels of EMG activation contrary to the effect of EMG on raw peak-to-peak amplitudes which followed expected trends that were reported in previous studies (Deriu et al., 2005).

### 4.1 The behavior of EMG was dependent upon the target level

The results of the present investigation showed that when subjects were asked to match a higher EMG target, they were more likely to produce EMG activation below the target level (Fig. 3, Fig. 4). Subjects were often within an acceptable range (+/- 10 μV) at lower EMG targets (Fig. 3, Fig. 4). Although some individuals were able to sustain higher levels of EMG activation (Fig. 5A and B), most were unable to do so (Fig. 2A and B). This tendency to “undershoot” the EMG target was more obvious at target levels above 50 μV (Fig. 2). It is also interesting to note that subjects had difficulty sustaining higher levels of EMG throughout the duration of the recordings (i.e., throughout all individual stimulus sweeps, Fig. 3A–D; Fig. 4A–D). These observations were consistent for the ipsilateral and contralateral muscles. To the best of our knowledge, this is the first report characterizing the behavior of activated masseter EMG across stimulus sweeps. Most reports investigating the effect of EMG on mVEMPs as well as the broader VEMP literature have reported a single average EMG value (Akin et al., 2004; McCaslin et al., 2014; Rosengren et al., 2019).

The tendency for EMG to fall below the EMG target level may be associated with jaw clenching to activate the masseter muscles. There is evidence that sustained muscle tension of the masseter could lead to muscle fatigue in as little as 20–30 s after clenching the jaw (Christensen, 1981). In addition, these findings may also be influenced by inter-subject variability in the level of maximum voluntary contraction (MVC) of the masseter muscle (Loi et al., 2020). Our study also showed that differences in EMG activation between sides can occur in some individuals (as shown in Fig. 5) and is consistent with individual data from other studies reporting EMG from clenched masseter muscles (Van Der Bilt et al., 2008).

Further, it is important to note that the behavior of EMG originating from the masseter muscles was similar to other types of muscles used to record VEMPs (e.g., cVEMPs). That is, there was greater inter-subject variability in mVEMP amplitudes observed at higher EMG targets, consistent with EMG behavior from other muscle groups (Akin et al., 2004; McCaslin et al., 2014).

### 4.2 EMG does not influence latency components

Our results suggest that mVEMP latency was not significantly impacted by varying levels of EMG activation. That is, regardless of the level of EMG, ipsilateral and contralateral p1/n1 latencies did not significantly change (Fig. 6). To the best of our knowledge, this is also one of the first reports describing invariance of mVEMP latencies across a range of EMG activation. More generally, the p1 and n1 latencies obtained in this study were comparable to other mVEMP studies presenting a tone burst stimulus (Vignesh et al., 2021).

### 4.3 EMG was associated with peak-to-peak amplitude

On average, raw peak-to-peak amplitude scaled with the underlying level of EMG activation and did not significantly differ between the ipsilateral and contralateral masseter muscles (Fig. 7). In addition, average EMG amplitude explained a significant portion of the variation in raw peak-to-peak amplitude (Fig. 8). These results were consistent with previous investigations of the effects of EMG on mVEMP (Deriu et al., 2005). These results further illustrate the inhibitory reflex properties shared with other types of VEMPs (e.g., cVEMP). Further, similar peak-to-peak amplitudes across the ipsilateral and contralateral sides provide additional evidence that the mVEMP is a true bilateral response (Fig. 1, Fig. 7).

Corrected peak-to-peak amplitude is often used as a measure of signal-to-noise ratio (SNR) and accounts for the effect of average EMG on raw peak-to-peak amplitude (McCaslin et al., 2014). This study showed that the impact of EMG on the response was reduced when corrected amplitudes were employed (Fig. 9) and were overall consistent with previous investigations reporting corrected mVEMP amplitudes (Vignesh et al., 2021). Additionally, mVEMP corrected amplitudes did not depend on EMG target level and were consistent with previous investigations in other types of VEMPs (McCaslin et al., 2014). Our results further demonstrated that average corrected amplitude asymmetry ratios were low (e.g., less than 13%) and did not depend on the EMG target level (see section 3.5). However, some of our subjects exhibited a large, corrected amplitude asymmetry percentage which could suggest a stronger vestibular projection to one side of the masseter muscle in some individuals.

Our results also demonstrated the vestibular component of the mVEMP (p1 amplitude) showed a strong relationship with peak-to-peak amplitude despite peak-to-peak amplitude also including the cochlear component of the response (n21). That is, at suprathreshold levels, there doesn't appear to be a difference between p1 amplitude and peak-to-peak amplitude. However, it is unclear if the relationship between p1 amplitude and peak-to-peak amplitude (p1-n1) changes at vestibular threshold or in the presence of disease.

### 4.4 Limitations/Future directions

The subject sample consisted of a group of young healthy individuals with the majority being female. It will be important in the future to examine the impact of age (i.e., recordings with an age stratified subject sample) on the recordability of the mVEMP greater variability of EMG activity is often observed in older adults (Akin et al., 2011). Also, the space, if any, that mVEMP occupies in the standard vestibular test battery will be determined in large part by the effect that well-defined pathologies have on the response.

## 5 Conclusions

In the present investigation we have reported most subjects were less likely to maintain EMG target levels higher than 50 μV suggesting that lower target levels may be more comfortable for the subject Additionally, there was no significant effect of tonic EMG activation on mVEMP latency while raw peak-to-peak amplitude scaled with the underlying level of EMG. On average, corrected mVEMP amplitude was unaffected by varied EMG targets and were similar between sides This study adds to the existing knowledge base on the effect that tonic EMG activity level has on the recordability of the mVEMP.

## Funding source

None.

## Declaration of competing interest

None.

## References

[bib1] Akin F.W., Murnane O.D., Panus P.C., Caruthers S.K., Wilkinson A.E., Proffitt T.M. (2004). The influence of voluntary tonic EMG level on the vestibular-evoked myogenic potential. J. Rehabil. Res. Dev..

[bib2] Akin F.W., Murnane O.D., Tampas J.W., Clinard C.G. (2011). The effect of age on the vestibular evoked myogenic potential and sternocleidomastoid muscle tonic electromyogram level. Ear Hear..

[bib3] Colebatch J.G., Halmagyi G.M., Skuse N.F. (1994). Myogenic potentials generated by a click-evoked vestibulocollic reflex. J. Neurol..

[bib4] Christensen L.V. (1981). Progressive jaw muscle fatigue of experimental tooth clenching in man. J. Oral Rehabil..

[bib5] de Natale E.R., Ginatempo F., Mercante B., Manca A., Magnano I., Ortu E. (2019). Vestibulo masseteric reflex and acoustic masseteric Reflex. Normative data and effects of age and gender. Clin. Neurophysiol..

[bib6] Deriu F., Podda M.V., Milia M., Chessa G., Sau G., Pastorino M. (2000). Masseter muscle activity during vestibular stimulation in man. Arch. Ital. Biol..

[bib7] Deriu F., Tolu E., Rothwell J.C. (2005). A sound-evoked vestibulomasseteric reflex in healthy humans. J. Neurophysiol..

[bib8] Deriu F., Ortu E., Capobianco S., Giaconi E., Melis F., Aiello E., Tolu E. (2007). Origin of sound-evoked EMG responses in human masseter muscles. J. Physiol..

[bib9] Deriu F., Giaconi E., Rothwell J.C., Tolu E. (2010). Reflex responses of masseter muscles to sound. Clin. Neurophysiol..

[bib10] Hickenbottom R.S., Bishop B., Moriarty T.M. (1985). Effects of whole-body rotation on masseteric motoneuron excitability. Exp. Neurol..

[bib11] Loi N., Manca A., Ginatempo F., Deriu F. (2020). The vestibulo-masseteric reflex and the acoustic-masseteric reflex: a reliability and responsiveness study in healthy subjects. Exp. Brain Res..

[bib14] McCaslin D.L., Fowler A., Jacobson G.P. (2014). Amplitude normalization reduces Cervical Vestibular Evoked Myogenic Potential (cVEMP) amplitude asymmetries in normal subjects: proof of concept. J. Am. Acad. Audiol..

[bib15] Mohammed Ali F., Westling M., Zhao L.H.L., Corneil B.D., Camp A.J. (2019). Splenius capitis: sensitive target for the cVEMP in older and neurodegenerative patients. Eur. Arch. Oto-Rhino-Laryngol..

[bib16] Portnuff C.D.F., Kleindienst S., Bogle J.M. (2017). Safe use of acoustic vestibular-evoked myogenic potential stimuli: protocol and patient-specific considerations. J. Am. Acad. Audiol..

[bib17] Rosengren S.M., McAngus Todd N.P., Colebatch J.G. (2005). Vestibular-evoked extraocular potentials produced by stimulation with bone-conducted sound. Clin. Neurophysiol..

[bib18] Rosengren Sally M., Colebatch J.G., Young A.S., Govender S., Welgampola M.S. (2019). Vestibular evoked myogenic potentials in practice: methods, pitfalls and clinical applications. Clinic. Neurophy. Pract..

[bib19] Van Der Bilt, Tekamp A., Van Der Glas H., Abbink J. (2008). Bite force and electromyograpy during maximum unilateral and bilateral clenching. Eur. J. Oral Sci..

[bib20] Vignesh S.S., Singh N.K., Rajalakshmi K. (2021). Tone burst masseter vestibular evoked myogenic potentials: normative values and test–retest reliability. J. Am. Acad. Audiol..

